# The physical and psychological aspects of quality of life mediates the effect of radiation‐induced urgency syndrome on disability pension in gynecological cancer survivors

**DOI:** 10.1002/cam4.6356

**Published:** 2023-07-24

**Authors:** Adnan Noor Baloch, Mats Hagberg, Sara Thomée, Gunnar Steineck, Helena Sandén

**Affiliations:** ^1^ Biostatistics, School of Public Health & Community Medicine, Institute of Medicine at Sahlgrenska Academy University of Gothenburg Gothenburg Sweden; ^2^ Occupational & Environmental Medicine, School of Public Health & Community Medicine, Institute of Medicine at Sahlgrenska Academy University of Gothenburg Gothenburg Sweden; ^3^ Department of Psychology University of Gothenburg Gothenburg Sweden; ^4^ Clinical Cancer Epidemiology, Department of Oncology, Institute of Clinical Sciences University of Gothenburg Gothenburg Sweden

**Keywords:** cancer survivorship, disability pension, early retirement, gynecological cancer, quality of life, return to work

## Abstract

**Background:**

Radiation‐induced fecal urgency syndrome is highly prevalent in gynecological cancer survivors. It is associated with decreased quality of life (QoL) and with disability pension. The literature remains unclear about the mediating role of physical and psychological aspects of QoL in the association between urgency syndrome and disability pension. Identifying the pathways between urgency syndrome and disability pension may help to create effective and timely interventions for increasing QoL and reducing disability pension among gynecological cancer survivors.

**Methods:**

We used patient‐reported outcome measures from working‐age gynecological cancer survivors (*n* = 247) and data on their disability pension from the official register. The mediating role of physical and psychological aspects of QoL was studied by utilizing mediation analysis based on the counterfactual framework, appropriate for binary outcome, binary mediator with an exposure–mediator interaction. The total effect (TE) was divided into direct and indirect effects using single mediation analysis. Adjusted relative risks and percentage mediated (95% confidence intervals) were calculated. All statistical tests were two‐sided.

**Results:**

Urgency syndrome increased the risk of disability pension both directly and indirectly (via QoL). Satisfaction with sleep mediated half of the TE (RR = 2.2 (1.1**–**4.1)) of urgency syndrome on disability pension. Physical health also mediated a similar proportion of the TE (RR = 2.1 (1.2–3.9)). The proportions mediated were higher for physical aspects of QoL (35%–71%) than for psychological aspects (2%–47%).

**Conclusions:**

The investigated aspects of the self‐assessed QoL of gynecological cancer survivors may play a role in these women's continuing work‐life. It appears that physical health, satisfaction with sleep, psychological well‐being, and other investigated aspects of QoL mediate the urgency syndrome–disability pension association.

## Graphical abstract

Quality of life (QoL) mediates the urgency syndrome and disability pension association among gynecological cancer survivors. Promoting QoL among gynecological cancer survivors may be an important approach to decrease their early exit from work‐life.

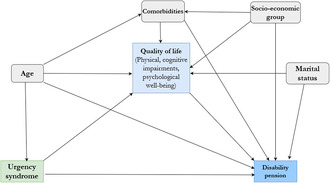

## 1 INTRODUCTION

The “pandemic of cancer treatment success”^1^ has resulted in a large number of gynecological cancer survivors. About half of these survivors are of working age,^2^ and the cancer and its treatment can alter their work‐life. Gynecological cancer survivors have been reported to have an increased risk of disability pension.^3^, ^4^, ^5^, ^6^ Carlsen et al. reported a 2.5 times higher risk of being awarded an early retirement pension for ovarian cancer patients compared to age‐matched controls, and a 1.3 times greater risk for cervical cancer patients compared to age‐matched controls.^4^ In a recent Norwegian study, gynecological cancer survivors had double the prevalence of disability pension compared with women in the general population.^6^ Radiotherapy to treat cancer in the pelvic area damages the bowel, which leads to a range of bowel symptoms and complications.^7^, ^8^, ^9^ While the survivors may be grateful for their recovery, they may also worry about the long‐term and lifelong radiation‐induced survivorship diseases. Women with a history of gynecological cancer experience radiotherapy‐induced gastrointestinal symptoms,^9^, ^10^ which impact their quality of life (QoL)^9^, ^11^, ^12^ and work ability.^3^, ^13^ Additionally, these symptoms cause anxiety and depression,^9^, ^12^ which can further worsen QoL. Earlier research has shown that gynecological cancer patients treated with adjuvant radiotherapy often report worse QoL (cognitive, physical, role, and social functioning) compared to those treated with surgery. Impairments or symptoms related to cancer treatment are reported to be barriers to a return to work.^14^ According to one study, important facilitators for returning to work include being able to control bowel function, being near to a toilet, and being able to take unscheduled breaks.^15^ Radiation‐induced fecal incontinence is reported to negatively impact physical QoL, psychological QoL, and social life of survivors.^9^, ^16^ In addition, radiation‐induced fecal urgency is suggested to impact everyday life.^17^

Using patient‐reported outcomes (PROMs) and factor analysis, Steineck et al.^18^ identified different syndromes of bowel dysfunction among gynecological cancer survivors. Urgency‐related symptoms such as *defecation urgency, defecation into clothing without forewarning, loose stools, and leakage of loose stools while awake* are highly prevalent among gynecological cancer survivors,^19^ and these are some of the symptoms included in the radiation‐induced urgency syndrome.

Return to work after a cancer diagnosis and treatment can be an important milestone for many working‐age survivors. It can be a sign of regaining control and resuming normalcy,^20^ improving physical, emotional, and mental well‐being. Work also provides financial stability as well as social connectedness, which can become disrupted during treatment. Given the increasing population of working‐age gynecological cancer survivors, we need better knowledge to prevent their early exit from the workforce. The pathways through which radiation‐induced urgency syndrome influences the likelihood that survivors go on disability pension are not known. It is important to identify *how* urgency syndrome affects the risk of disability pension. By identifying and examining the mediating pathways, one can determine the direct and indirect impact of urgency syndrome on disability pension. This can help with the development of interventions that target the different mediators and improve work‐related outcomes for affected women. While it may be hard to eliminate all adverse effects of radiation‐induced urgency syndrome, addressing the mediating factors is vital in preventing urgency syndrome from having a large negative influence on the likelihood of disability pension. Increased knowledge about such potentially modifiable mediators may help prevent at least some early retirement due to disability in this group of patients, whose problems have long been overlooked. To prevent undesirable outcomes of radiation‐induced urgency syndrome, it would be worthwhile to explore every avenue.

The self‐rated QoL provides vital information on an individual's physical, psychological, and social dimensions of functioning and well‐being. It encapsulates the patient's subjective health state and is used to evaluate the treatment outcomes. In research, QoL is commonly used as a pseudo measure of self‐rated health. Previous research reports the associations between self‐rated health and disability pension as well as physical QoL and disability pension.^21^, ^22^, ^23^ Cancer survivors with fewer chronic health conditions at baseline report a greater increase in physical and mental QoL at their 5‐year follow‐up.^24^ Cancer patients often experience depression and anxiety.^25^ An earlier study^26^ reports more depressive symptoms among gynecological cancer survivors than their age‐matched controls. Cancer changes the patient's self‐image, daily activities, interpersonal relationships, and role functioning. In addition, radiation‐induced urgency symptoms negatively affect psychological and social QoL while hindering maintenance of daily functions.^16^ The influential Cancer and work model^27^ identifies various aspects of function, including physical, cognitive, emotional, and interpersonal function, as mediators of treatment‐related symptoms and work‐related outcomes in cancer survivors. Another systematic review reported that health impairments (physical symptoms and comorbid disease) and psychosocial factors (depression, anxiety, etc.) influence employment in cancer survivors.^28^

Hence, given this background, we hypothesize the physical and psychological aspects of QoL to mediate the urgency syndrome–disability pension association. We expect urgency syndrome to have a negative influence on physical and psychological aspects of QoL, which in turn negatively influence survivors' work ability. We investigated and quantified the mediating role of physical and psychological aspects of QoL on radiation‐induced urgency syndrome and future disability pension association.

## 2 METHODS

Previous studies have described the recruitment and data collection process in detail,^16^, ^18^, ^19^ but the following is a summary.

### 2.1 Study design and patients

This was a register‐based study. In 2006, cancer survivors answered a postal questionnaire. Patient‐reported data were used as mediator variables and to build our urgency syndrome predictor. The disability pension data for the year 2008 was taken from the Swedish register, Longitudinal Integrated Database for Health Insurance and Labour Market Studies (LISA).

All females (*n* = 1800) treated with external beam radiation therapy during 1991–2003 at Sahlgrenska University Hospital or at Karolinska University Hospital in Sweden were initially selected. The inclusion criteria of being able to read and understand Swedish, having been born in 1927 or later, and not having had recurrence of cancer were satisfied by 823. As a control group, 486 women matched on age and place of residence were recruited from the general population. Among the controls, eight who previously had radiotherapy to the pelvic region or who were unable to read and understand Swedish were excluded. The recruitment process is outlined in detail in the flowchart (Figure S1).

### 2.2 Study procedures

In 2006, the 823 cancer survivors and 478 controls were mailed a letter that explained the research objectives and were thereafter telephoned by a neutral third‐party secretariat. Most of the study group (89% of survivors and 88% of controls) gave informed consent during the telephone call and then received a paper questionnaire sent by post. They were sent a postal reminder after 3 weeks and also received a reminder phone call. Altogether, 650 (89%) survivors responded to the questionnaire; of these, 7 were excluded due to missing data on syndromes, 7 due to a missing social insurance number, 363 because they were ≥65 years old (in 2008), and 20 because they had a bowel stoma. In total, 403 survivors were excluded, leaving 247 survivors for inclusion (Figure S1). In Sweden, only individuals 19–64 years old can receive disability pension.^21^ Among the controls, 344 responded to the questionnaire.

Data on demographics and symptoms from the gastrointestinal tract, genitals, legs, pelvic bones, abdomen, and urinary bladder were collected through the postal questionnaire, which also included questions on comorbidities, psychological issues, QoL, and sexual function. Study participants were asked to report the intensity and duration of their symptoms. The development and validation of the scales used in the postal questionnaire followed established practices and have been documented in >80 articles.^16^, ^19^, ^29^, ^30^ The questionnaire was developed using in‐depth interviews with 26 gynecological cancer survivors treated with pelvic radiotherapy. The study questionnaire was validated using face‐to‐face validity and was afterward revised based on comments from 20 gynecological cancer survivors. It was then tested in a pilot study with 20 other gynecological cancer survivors. Details of the development and validation of the postal questionnaire have been reported elsewhere.^19^ The indicators of psychological QoL, anxiety and depression, were previously regarded as valid and sensitive.^31^, ^32^

### 2.3 Urgency syndrome (predictor)

The patient‐reported gastrointestinal symptoms were analyzed using the modified exploratory factor analysis approach described by Steineck et al.^18^ The 28 gastrointestinal symptoms were found to be linked to six “factors” and were interpreted as *radiation‐induced survivorship syndromes*.^18^ For five of the six syndromes (all except *constipation*), the factor score quantiles showed a statistically significant difference between the survivors and the controls. The survivors were classified as having a syndrome if their factor loading (syndrome intensity) on that syndrome was greater than the 95th percentile of the controls.^18^ The same cancer survivor could be categorized as having more than one syndrome. Based on previous findings^16^, ^19^ from our research unit, urgency‐related symptoms such as defecation urgency and defecation into clothing without forewarning, among others, had the largest age‐adjusted risk differences and/or age‐adjusted relative risks between cancer survivors and controls. Furthermore, defecation into clothing without forewarning was reported to negatively influence cancer survivors' global QoL, social QoL, and work ability. As a result, we opted to study only the mediators of urgency syndrome on disability pension. Gastrointestinal symptoms included in urgency syndrome are provided in the supporting information, Appendix S1.

### 2.4 Disability pension (outcome)

By using the survivor's personal identity number, the data on disability pension were obtained from the LISA. This database is a compilation of existing data on individuals from different official Swedish registries. All individuals aged ≥16 years, registered in the Total Population Register as of December 31st each year and covered by the social insurance system, are included in the LISA.^33^

In the Swedish social insurance system, individuals can receive disability pension if they are 19–64 years old, have their work capacity diminished by at least one‐fourth due to an illness or disability, are unable to work for at least 1 year in any work in the entire labor market, and are insured in Sweden. The social security system only includes individuals legally living and working in Sweden. An insurance physician assesses the individual and submits a medical statement describing the illness and the impairments in work capacity. Students, unemployed individuals, and homemakers are also eligible for disability pension. An individual who is at least 62 years old can combine disability pension with early old‐age pension. The outcome *disability pension* used in this study was binary: the pension was either “granted” or “not granted.”

### 2.5 Quality of life (mediator)

The physical and psychological aspects of QoL were self‐assessed and measured using single‐item 7‐point visual digital scales, previously demonstrated to have good correlation with established instruments.^31^ A sample question is, “How would you evaluate your quality of life during the past 6 months?” with answer alternatives ranging from 1 = “no quality of life” to 7 = “the best possible quality of life.” The items were dichotomized according to a median value into “low to moderate” [1–5] versus “high” [6, 7]. However, items measuring depression and anxiety were dichotomized into “low to moderate” [1–3] versus “high” [4–7]. The QoL questions and answer alternatives are provided in the supporting information (Appendix S2).

### 2.6 Mediation analysis framework

Mediation analysis is a regression analysis with a complex dependency structure. This method is used to extricate the pathways linking an exposure to an outcome. Furthermore, it is used to estimate the relative size of different pathways through which an exposure (X) may affect an outcome (Y). Traditional methods of mediation analysis^34^ may fall short when dealing with binary outcomes or binary mediators, as well as interactions between the effects of the exposure and the effects of the mediator. If the exposure increased vulnerability to the effect of the mediator, an interaction would be present. Inclusion of exposure–mediator interactions often increases the size of indirect effects, as a result increasing the power to detect mediated effect and allows for additional model flexibility.^35^ Hence, exposure–mediator interactions should be included by default and should only be excluded if they do not seem to change the estimates very much.^35^, ^36^ We employed the counterfactual‐based mediation analysis approach^36^, ^37^ to mitigate all the mentioned concerns. This approach assumes adjusting for the confounding variables of (a) the exposure and the outcome association, (b) the mediator and the outcome association, (c) the exposure and the mediator association. In addition, (d) no direct effect of the exposure that could confound the mediator and the outcome association. Assumptions (a‐d) also require an assumption of temporal ordering. Sensitivity analysis technique can be employed to assess the robustness of results in the presence of unmeasured confounding.^38^ This technique allows for an assessment of the combined effect of the confounder‐outcome association and the confounder–mediator association that would be necessary to account for or “explain away” indirect or direct effect.^38^

**FIGURE 1:**
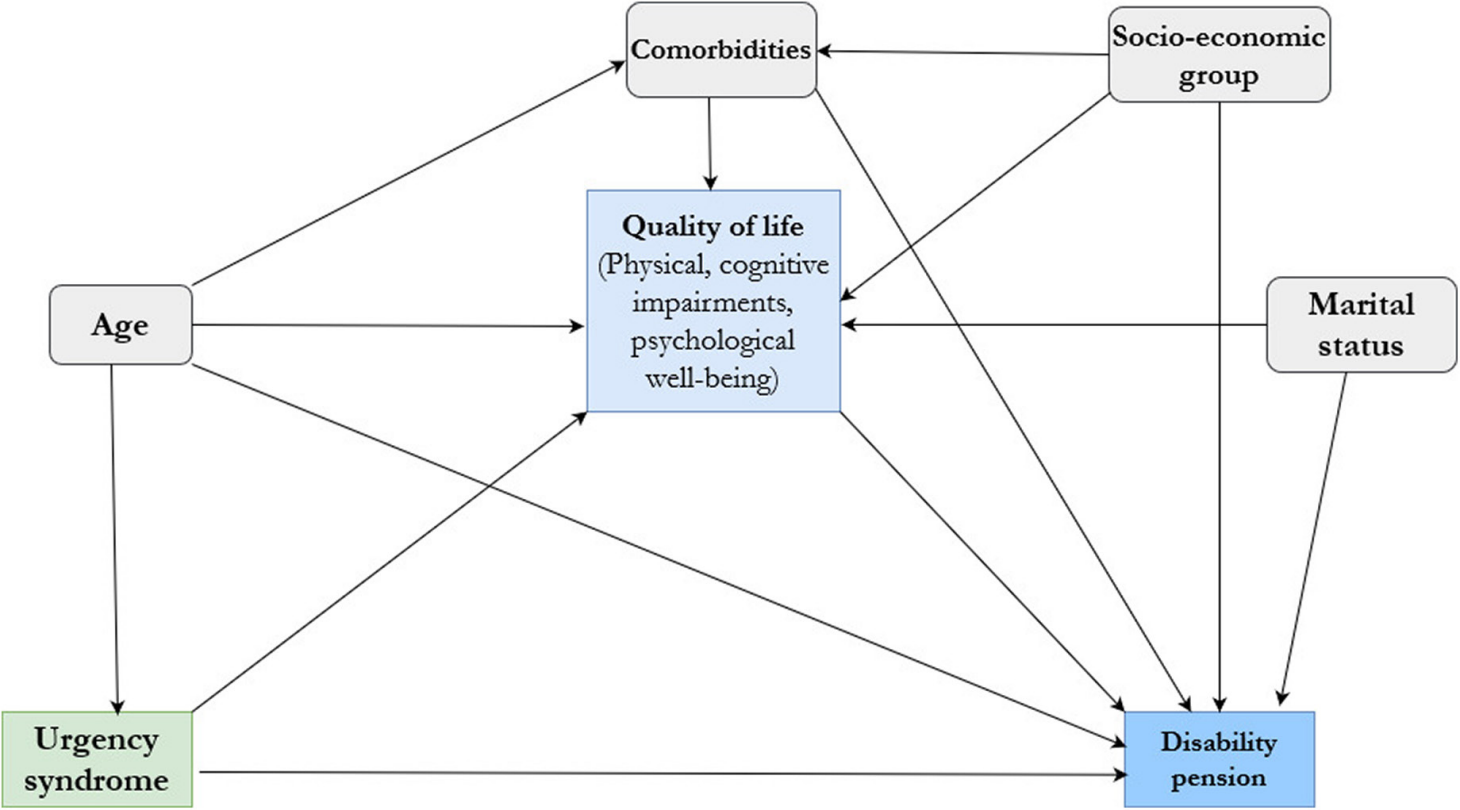
Direct acyclic graph representing the causal model.

Based on the previous research findings (detailed in the Introduction), subject‐matter knowledge, and by integrating the cancer and work model,^27^ a causal model (Figure 1) was developed to estimate the mediation effects by applying directed acyclic graph (DAG). The following confounders were incorporated in the causal DAG; age at baseline (in years), marital status at baseline, socioeconomic group (an occupation‐based derived variable obtained from the national register LISA.^33^ It was built on the model developed by Eurostat working group to create the European socioeconomic classification^39^) and number of self‐reported comorbidities. Survivors were classified as having none, one, two, and three or more comorbidities based on sum of self‐reported chronic conditions as diabetes, hypertension, cardiac infarction, angina pectoris, heart failure, Crohn's disease, ulcerative colitis, bowel disease, IBS, hemorrhoids, prolapse, neurological disease, psychological disorder, joint disorder, rheumatism, kidney disease, lung disease, thrombosis, and osteoporosis.

The total effect (TE) of binary urgency syndrome (exposure) on binary disability pension (outcome) was decomposed into the so‐called “natural direct effect (NDE)”—the influence of the urgency syndrome unexplained by the mediator—and the “natural indirect effect (NIE)”—the influence of urgency syndrome that is explained by the same mediator.^36^, ^37^ These effects are defined both for rare binary outcomes (≤10%) on the odds ratio scale and for common binary outcomes on the risk ratio scale.^36^, ^37^ The proportion of the effect mediated (PM) is a measure of the importance of the pathway via that mediator in explaining the association between exposure and the outcome. The effects (total, direct, and indirect) were computed on risk ratio scale (adjusted relative risks) (RRs) and proportion of effect mediated (PM, on risk difference scale) along with their respective bias‐corrected bootstrap 95% confidence intervals (CI).These effects were calculated by using an aspect of self‐assessed QoL as a mediator (M) for the association between urgency syndrome(X) and disability pension(Y), including an urgency syndrome‐mediator (XM) interaction, while without adjusting for another mediator/‐s (Figure 1). The proportion of effect mediated (95% CI) was the main effect of interest in this paper.

### 2.7 Statistical analysis

The AGReMA Checklist^40^ was followed (Checklist S1). Frequency counts and percentages were used to describe categorical variables. Not having urgency syndrome and having high QoL (low depression and anxiety) were used as the reference category and adjusted relative risk of being awarded a disability pension was modeled. Level of significance was set at 5%, and all statistical tests were two‐sided. SAS CAUSALMED procedure (using SAS 9.4 statistical analysis software) and R‐package “mediation” (using RStudio v2022.07.2 Build 576 “Spotted Wakerobin”, RRID:SCR_000432) were used for the mediation analysis. All other statistical analyses were performed using SAS9.4 statistical analysis software (SAS Institute, RRID:SCR_008567).

### 2.8 Sensitivity analysis

We computed mediational E‐values to assess the potential impact of unmeasured confounding on our observed mediational effects.^38^ These E‐values were calculated specifically for the adjusted point estimates of the natural indirect effects. An additional sensitivity analysis to remove the negative influence of earlier disability pension (pre‐2006) on self‐reported QoL in 2006 was also performed. Gynecological cancer survivors (*n* = 60) who received disability pension during 2004–2006 were excluded (Table S1). The data that met this rigorous inclusion criterion were reanalyzed, and results are reported in Table S2. Mediation analysis using earlier disability pension as a covariate was not possible due to zero cell counts.

## 3 RESULTS

Among all gynecological cancer survivors, 91 (37%) had urgency syndrome (at baseline) and 66 (27%) survivors were receiving disability pension (at follow‐up). At follow‐up, survivors suffering from urgency syndrome and on disability pension had a mean age of 58 years with a 95% CI of 56–60. Among this group, the most common diagnosis was endometrial cancer (51%), followed by cervical (26%), ovarian (9%), fallopian tube (6%), vaginal (3%), sarcoma uteri (3%), and vulvar (3%) cancer. Baseline clinical and background data of the survivors are provided in Table 1, and the descriptive statistics for the mediator variables for all 247 gynecological survivors are provided in Table 2.

**TABLE 1:** Baseline (in 2006) clinical and demographic data for all gynecological cancer survivors, and separately for those with and those without urgency syndrome.

	All survivors		Urgency syndrome			
			Yes		No	
	Counts	%	Counts	%	Counts	%
	247	100	91	37	156	63
Age						
16–29 years	2	1	2	2	0	0
30–49 years	66	27	24	26	42	27
50–64 years	179	72	65	71	114	73
Marital status						
Married/living with partner	160	65	58	64	102	66
Widow	12	5	5	5	7	5
Has partner but lives alone	21	9	11	12	10	6
Single	53	22	17	19	36	23
Not stated	1				1	
Self‐reported employment						
Retired	4	2	0	0	4	3
Student	5	2	2	2	3	2
Sickness absence	9	4	7	8	2	1
Housewife, other	10	4	3	3	7	5
Unemployed	12	5	3	3	9	6
Disability pension	35	14	22	24	13	8
Employed	169	68	54	59	115	74
Not stated	3	1	0	0	3	2
Socioeconomic group (occupation‐based)						
Senior officials and managers	16	6	7	8	9	6
Qualified officials	53	21	20	22	33	21
Other white‐collar workers	44	18	11	12	33	21
Small business owners excluding farmers	8	3	2	2	6	4
Professionals in trade, service and care occupations	45	18	18	20	27	17
Skilled workers	2	1	1	1	1	1
Other workers	20	8	6	7	14	9
Not economically active	59	24	26	29	33	21
Diagnosis						
Sarcoma uteri	9	4	3	3	6	4
Vulvar cancer	2	1	1	1	1	1
Vaginal cancer	11	4	4	4	7	4
Cervical cancer	93	38	33	36	60	38
Endometrial cancer	104	42	38	42	66	42
Ovarian cancer	23	9	10	11	13	8
Fallopian tube cancer	5	2	2	2	3	2
Treatment modality						
Surgery + EBRT	18	7	8	9	10	6
Surgery + EBRT + BT	110	45	35	38	75	48
Surgery + EBRT + chemo	28	11	14	15	14	9
Surgery + EBRT + BT + chemo	56	23	18	20	38	24
EBRT	1	<1	1	1	0	0
EBRT + BT	11	4	5	5	6	4
EBRT + chemo	5	2	5	5	0	0
EBRT + BT + chemo	17	7	5	5	12	8
Not stated	1				1	1
Number of comorbidities^a^						
No comorbidities	108	44	26	29	82	53
Have 1 comorbidity	47	19	21	23	26	17
Have 2 comorbidities	43	17	20	22	23	15
Have atleast three comorbidities	48	20	24	26	24	15
Not stated	1				1	
Parity						
Never given birth	80	32	27	30	53	34
1–3 children	151	61	56	62	95	61
>3 children	16	6	8	9	8	5

Abbreviations: BT, brachy therapy; chemo, chemotherapy; EBRT, external beam radiation therapy.

^a^ Sum of self‐reported chronic conditions as diabetes, hypertension, cardiac infarction, angina pectoris, heart failure, Crohn's disease, ulcerative colitis, bowel disease, IBS, hemorrhoids, prolapse, neurological disease, psychological disorder, joint disorder, rheumatism, kidney disease, lung disease, thrombosis, and osteoporosis.

**TABLE 2:** Descriptive statistics for the mediator variables, physical and psychological aspects of quality of life (QoL), for all gynecological cancer survivors.

Self‐assessed QoL		Median (first–third quartile)	Counts (%)^a^
Global QoL	Low to moderate	5 (4–6)	140 (57)
Physical aspects			
Global physical health	Low to moderate	5 (4–6)	167 (68)
Physical strength (condition)	Low to moderate	5 (4–6)	165 (67)
Psychological aspects			
Satisfied with sleep	Low to moderate	4 (3–6)	123 (50)
Satisfied with concentration	Low to moderate	5 (4–6)	152 (62)
Satisfied with memory	Low to moderate	5 (4–6)	149 (61)
Psychological wellbeing	Low to moderate	5 (4–6)	167 (68)
Self‐esteem	Low to moderate	5 (4–6)	146 (59)
Having meaning in life	Low to moderate	6 (4–7)	175 (71)
Worry or anxiety	High^b^	3 (2–5)	105 (43)^b^
Depressed or feeling sad	High^b^	3 (2–5)	105 (43)^b^

^a^ This shows the frequency and the percentage of survivors scoring ≤ median.

^b^ For worry or anxiety and depressed or feeling sad, the frequency and the percentage of those reporting values higher than the median.

The urgency syndrome‐mediator interactions were only evident for global Qol, physical health, physical strength, psychological well‐being, and satisfaction with sleep. Moreover, inclusion of exposure–mediator interactions increased the indirect effects for these mediators (Table 3; Table S4). All adjusted TEs, almost half of the adjusted direct effects (NDEs), indirect effects (NIEs), and their 95% CI were >1, increasing the likelihood of disability pension. Our analysis showed similar magnitude for all effects (direct, indirect, and total) for most of the investigated mediators (Table 3). The patterns of all effects were similar for all self‐reported physical and psychological aspects of QoL. Some aspects of self‐reported QoL were more important (higher PM) than others in explaining the association between radiation‐induced urgency syndrome and disability pension (Table 3). For example, about 46% of the TE (RR = 2.3 (1.1–3.7)) of urgency syndrome on disability pension was mediated through global physical health. However, the 95% CI for the proportion mediated was 22%–110%. In the same manner, 35% (7%–64%) of TE (RR = 2.1 (1.2–3.9)) obtained from a single mediation analysis was mediated via physical strength, 47% (18%–111%) via satisfaction with sleep, and 43% (15%–71%) via psychological well‐being. A larger proportion of effect was mediated (PM) via the global and physical aspects of QoL (between 35% and 71%) compared with the psychological aspects of QoL (between 2% and 47%) (Table 3).

**TABLE 3:** Adjusted NDE, NIE, and TE of radiation‐induced urgency syndrome on disability pension in presence of exposure–mediator interaction (*n* = 247 gynecological cancer survivors). Data on disability pension were obtained from the official register; the table shows the findings of mediation analysis while adjusting for age (in years), marital status, occupation‐based socioeconomic group, and number of comorbidities.

Mediator (M)	Adjusted relative risk^a^ (95% CI)			Proportion mediated^b^
	NDE	NIE	TE	(95% CI)
Global QoL	1.3 (0.8–2.4)	**1.6 (1.3–2.0)**	**2.1 (1.2–3.7)**	**71% (40%–163%)**
Physical aspects				
Global physical health	1.7 (0.8–2.8)	**1.3 (1.0–1.6)**	**2.3 (1.1–3.7)**	**46% (22%–110%)**
Physical strength (condition)	1.7 (0.9–3.4)	**1.2 (1.0–1.4)** ^c^	**2.1 (1.2–3.9)**	**35% (7%–64%)** ^c^
Psychological aspects				
Satisfied with sleep	**1.6 (0.8–3.1)**	**1.3 (1.1–1.7)**	**2.2 (1.1–4.1)**	**47% (18%–111%)**
Psychological wellbeing	**1.7 (1.0–2.5)** ^c^	**1.3 (1.1–1.6)**	**2.3 (1.4–3.2)** ^c^	**43% (15%–71%)** ^c^
Satisfied with concentration	**1.5 (1.0–2.3)** ^c^	1.2 (1.0–1.4)^c^	**1.8 (1.0–2.6)**	**36% (2%–70%)**
Self‐esteem	**1.9 (1.1–5.8)**	1.2 (1.0–1.3)^c^	**2.2 (1.3–5.0)**	**27% (1%–52%)**
Worry or anxiety	1.6 (0.8–2.7)	1.1 (0.9–1.4)	1.8 (0.9–2.9)	20 (−13%–103%)
Having meaning in life	1.7 (0.9–3.7)	1.1 (1.0–1.3)	**1.8 (1.0–4.0)**	20% (−12%–93%)
Satisfied with memory	**1.9 (1.0–2.8)**	1.1 (0.8–1.3)	**2.0 (1.1–2.7)**	12% (−32%–49%)
Depressed or feeling sad	**2.2 (1.3–4.5)**	1.0 (0.9–1.3)	**2.3 (1.4–4.5)**	2% (−17%–74%)

Abbreviations: CI, confidence interval; NDE, natural direct effect; NIE, natural indirect effect; QoL, quality of life; TE, total effect.

*Note*: Estimates of natural direct effect, indirect effect, total effect, and proportion of effect mediated were obtained by using an aspect of self‐assessed QoL as a mediator(M) for the association between urgency syndrome(X) and disability pension(Y) in presence of XM‐interaction. **Bold numbers** indicate a statistically significant effect at 5% level of significance. **NDE** = the contrast between the counterfactual outcome while being exposed and the counterfactual outcome while the same individual was not exposed, mediator assuming value it would have taken while being not exposed. **NIE** = the contrast, having set the *exposure = Yes, exposed* between the counterfactual outcome (mediator assumed whatever value it would have taken at a value of the *exposure = Yes, exposed* and the counterfactual outcome if the mediator assumed whatever value, it would have taken at a reference value of the *exposure = not exposed*).

^a^ Adjusted relative risk with bootstrap bias corrected 95% CI.

^b^ Proportion mediated = NDE*(NIE‐1)/(NDE*NIE‐1), with bootstrap bias corrected 95% CI.

^c^ Wald 95% CI.

The sensitivity analysis of unmeasured confounding using mediational E‐values suggested that most of the mediators played a significant role in explaining the association, reducing the potential impact of unmeasured confounding on the observed association (Table S3). The likelihood of an unmeasured confounding factor explaining the association, rather than the mediator, was considered to be low for the mediators with an E‐value ≥ 1.6 observed in our analysis (all but for “satisfied with memory,” having a meaning in life, worry or anxiety, and depressed/feeling sad).

The sensitivity analysis after removing the gynecological survivors who had received disability pension during 2004–2006 resulted in a sample size of *n* = 187, with seven cases of disability pension at the follow‐up in 2008 (Table S1). Having only seven cases was insufficient for any but the large effects; there may have been effects, but the number of events was too small for detecting any effect sizes of interest to us (RR ≥ 2). The results from both mediation analyses (lenient inclusion criterion with *n* = 247 and the rigorous inclusion criterion with *n* = 187) gave indications in the same direction. This applies to all effects (>1), increasing risk of impaired QoL and disability pension among gynecological cancer survivors with urgency syndrome. However, PROC CAUSALMED returned abnormally wide CI for the rigorous inclusion criterion. While the indirect effects were almost same, the total and direct effects were stronger in the latter mediation analysis (Table 3; Table S2).

## 4 DISCUSSION

Linking self‐reported and register data, we discovered that various aspects of QoL mediated the association between radiation‐induced urgency syndrome and disability pension. This suggests that, in gynecological cancer survivors suffering from radiation‐induced urgency syndrome, QoL is important for their continuing work‐life.

It is difficult to compare our findings with earlier studies. The mediating role of physical and psychological aspects of QoL among gynecological cancer survivors has not been previously investigated, although the association between side effects of radiotherapy and QoL,^8^, ^16^ and between QoL, self‐rated health, and disability pension,^21^, ^22^, ^23^ have been investigated. Our conclusions agree with earlier studies that separately looked at these two associations.

In our investigation, we found a larger mediating role of global Qol and physical aspects of QoL than the psychological aspects of QoL between radiation‐induced urgency syndrome and disability pension. In other words, the radiation‐induced urgency syndrome influenced these aspects of QoL. This in turn influenced the likelihood of early retirement due to disability among gynecological cancer survivors but with a different magnitude. Earlier investigations found that gynecological cancer treatment can precipitate a variety of symptoms, especially gastrointestinal and psychosocial ones, that may adversely affect survivors' QoL.^8^, ^10^, ^11^, ^12^ Gami and colleagues^41^ interviewed 107 cancer patients (65 gynecological) treated with pelvic radiotherapy. Of the gynecological cancer patients, 57 reported gastrointestinal symptoms and 35 of these patients reported that the symptoms affected their QoL. The multicenter randomized PORTEC‐1 trial reported increased bowel symptoms and lower physical and role‐physical functioning (SF‐36) in gynecological cancer patients treated with radiotherapy compared with no additional treatment.^42^ These findings are consistent with our results, that is, urgency syndrome negatively affecting QoL.

In addition, the negative effect on QoL is associated with reduced work ability. Our study found that the mediators explain part of the association between the syndrome and future disability pension. This means that worsening of QoL explains a portion of the urgency syndrome–disability pension association.

In a large follow‐up study of employed individuals, Haukenes et al.^21^ found that the physical component of QoL is a stronger predictor of disability pension than the mental component. This was independent of age, gender, education, occupational class, smoking, physical activity, and BMI. In concordance with this study, we found a smaller mediating role of psychological aspects of QoL than the physical aspects of QoL. This is an important finding, and one can hypothesize that psychological aspects appear to have less impact than the physical aspects considering the mean age of our survivors (58 years). A study by Månsson et al.^43^ found that individuals with less than perfect self‐reported health are about four times more likely to receive disability pension. Dunberger et al. revealed that fecal incontinence negatively affected gynecological cancer survivors' QoL and ability to work.^16^

### 4.1 Strengths and limitations of the methodology used

We used official records on disability pension and a validated questionnaire for PROMs. Hence, there is no attrition bias and reduced interviewer bias. We believe that no previous study has investigated the mediating role of QoL in the association between radiation‐induced urgency syndrome and future disability pension. Therefore, this study explores a new way of looking at the role of reporting urgency syndrome at baseline and disability pension 2 years later.

The classic regression approach^34^ is commonly utilized in research, but the decomposition of TE into direct (NDE) and indirect effect (NIE) would not uniformly work for binary mediators, binary outcomes, and for exposure–mediator interactions. Current advancements in causal inference offer new methods for addressing the abovementioned shortcomings in the classic approach while highlighting the assumptions required for identification. In addition, we conducted sensitivity analysis to gauge the potential influence of unmeasured confounding on the nullification of our indirect effects. This evaluation involved the calculation of mediational E‐values. One of our major strength lies in the innovative utilization of recently developed advanced methods for mediation analysis. There is no hint of systematic dropout in the recruitment process that can influence our findings. Furthermore because of the close cooperation with the survivors during the questionnaire development phase, we judge the possible misclassification of the urgency syndrome and QoL to be low. If the survivors answering the questionnaire have had better health and QoL than the survivors who died before they could answer the questionnaire, then it is possible that the effects have been underestimated.

Several other factors, for example earlier disability pension, functional status in relation to work demands, or the work environment, may also influence the association between urgency syndrome and going on disability pension. We are aware that type of cancer may affect both QoL and the risk of receiving a disability pension, but the purpose of this study was to look at what mediates the increased risk of receiving a disability pension in those with radiation‐induced urgency syndrome, and in that analysis, we do not believe that type of cancer is a confounder. Moreover, due to the nature of Swedish social insurance systems, our research findings are mostly applicable to similar contexts. Thus, care should be taken in consideration of our findings. Single‐item questions may not completely capture complex constructs compared with multi‐question scales, but they yield meaningful information. The questionnaire development method described in this and earlier articles^16^, ^19^, ^29^, ^30^ provides a high validity and the rationale^30^ for using a single‐item question instead of a summary score scale in cancer survivors. Our mediation analysis has been underpowered to detect statistically significant effects for some mediator. We could not distinguish between partial and full‐time disability pension. In addition, it should be noted that findings from this study may be considered exploratory rather than descriptive or explanatory. However, the sensitivity analysis did not make any large, notable changes in our interpretation of the results.

### 4.2 Implication

There is a need to develop interventions that address these mediators and suit the needs of gynecological cancer survivors. This knowledge serves as a foundation for developing future studies and targeted interventions and policy recommendations that directly address the identified mediators. Ultimately, this approach enhances the potential for meaningful and impactful changes in the real world. For example, some survivors may benefit from physical therapy to improve physical health and/or strength, while others may benefit from counseling to address overall psychological well‐being or improve sleep. Even for survivors who suffer from urgency syndrome, there may be possibilities to sustain work ability. In summary, more and better research is crucial in identifying interventions that can improve the gastrointestinal health and QoL for gynecological cancer survivors. By addressing the physical, psychological, and social challenges that survivors face, these interventions can help survivors live healthier and more fulfilling lives.

## 5 CONCLUSION

The investigated aspects of the self‐assessed QoL of gynecological cancer survivors may play a role in these women's continuing work‐life. It appears that physical health, physical strength, satisfaction with sleep, psychological well‐being, and other investigated aspects of QoL mediate the urgency syndrome–disability pension association.

## AUTHOR CONTRIBUTIONS

**Adnan Noor Baloch:** Conceptualization (equal); data curation (lead); formal analysis (lead); investigation (equal); methodology (lead); resources (equal); software (lead); validation (lead); visualization (lead); writing – original draft (lead); writing – review and editing (lead). **Mats Hagberg:** Conceptualization (equal); funding acquisition (equal); investigation (equal); resources (supporting); supervision (equal); writing – review and editing (supporting). **Sara Thomée:** Conceptualization (equal); investigation (equal); writing – review and editing (supporting). **Gunnar Steineck:** Conceptualization (equal); funding acquisition (supporting); investigation (equal); resources (equal); writing – review and editing (supporting). **Helena Sandén:** Conceptualization (equal); investigation (equal); project administration (lead); supervision (equal); writing – review and editing (supporting).

## FUNDING INFORMATION

This work was supported AFA Försäkring (#180031 PI Mats Hagberg) and by FoU‐Västra Gotalandsregionen (#233051 Professor Gunnar Steineck). The funder had no role in study design, data collection, analyses, or any other stage of this study.

## CONFLICT OF INTEREST STATEMENT

The authors of this manuscript have no conflicts of interest.

## ETHICS STATEMENT

This study has been approved by the Regional Ethical Review Board in Gothenburg, Sweden (dossier. no. 671‐17) and conforms to the provisions of the Declaration of Helsinki. All study participants gave consent to participate. This study was registered with ClinicalTrials.gov (Identifier: NCT03961217) (https://clinicaltrials.gov/ct2/show/NCT03961217).

## Supporting information


Figure S1.
Appendix S1.
Appendix S2.
Checklist S1.
Table S1.
Table S2.
Table S3.
Table S4.

## Data Availability

Information on health is sensitive, and such data cannot be shared without approval from the Swedish Ethical Review Authority. Contact the corresponding author for more information and code for mediation analysis. Data on disability pension were provided by a third party (Statistics Sweden), which prohibits authors from sharing data. For more information, contact Statistics Sweden, Box 24300, 10451 Stockholm, Sweden.
