# Supplementary material for: The physical and psychological aspects of quality of life mediates the effect of radiation‐induced urgency syndrome on disability pension in gynecological cancer survivors
Source: Cancer Med. 2023 Jul 24;12(16):17377–88. doi: 10.1002/cam4.6356 (PMC10501287; doi:10.1002/cam4.6356)
Supplement: Supplementary file 1 — Figure S1. Appendix S1. Appendix S2. Checklist S1. Table S1. Table S2. Table S3. Table S4. [file CAM4-12-17377-s001.pdf]

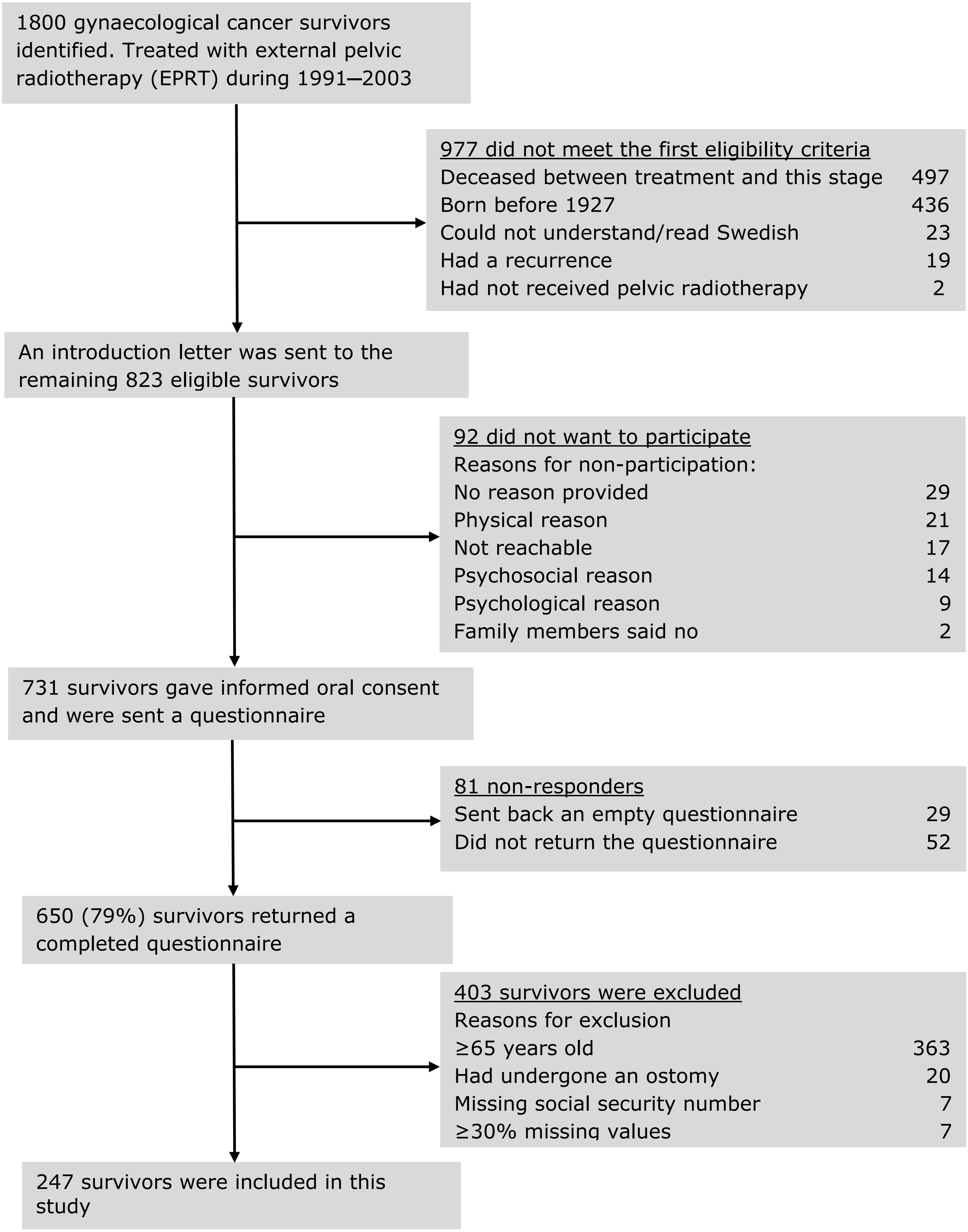

Figure S1: Flowchart of recruitment and selection of gynecological cancer survivors

Appendix S1: Self-reported gastrointestinal symptoms included in urgency syndrome and their estimated factor loading (symptom intensity)

Factor loading	English translation	Original Swedish text
0.85	Sudden defecation urgency requiring lavatory	Har Du haft plötsligt påkomna avföringsträngningar toalettbesök
0.74	Immediate need to defecate	Har Du haft omedelbart behov av en toalett om Du behövt
0.71	Loose stools	Har Du haft lös avföring
0.69	Inability to hold stools for >5 minutes during urgency	Hur länge har Du kunnat hålla avföringen vid trängningar
0.61	Need to repeat defecation within one hour	Har Du återvänt till toaletten inom en timme efter avföring for att tömma"
0.40	Leakage of loose stools while awake	Har Du haft läckage av lös avföring när Du varit vaken"
0.35	Fecal leakage without warning despite pre-emptied bowel	Har Du utan förvarning läckt avföring i kläderna
0.34	Abdominal pain	Har Du haft smärtor i buken
0.32	Abdominal bloating	Har Du haft känsla av uppblåsthet i magen
0.27	Involuntary foul smelling flatulence	Har Du haft illaluktande gasavgångar som Du inte kunnat stoppa
0.26	Defecation into clothing without forewarning	Har Du utan förvarning tomt all avföring i kläderna
0.25	Involuntary unspecified flatulence	Har det hänt att Du inte har kunnat hålla kvar gas"
0.23	Anal itching	Har Du haft klåda vid ändtarmsöppningen, det senaste halvåret?
0.22	Leakage of loose stools while asleep	Har Du haft läckage av lös avföring, när Du sovit"
0.22	Anal pain	Har Du haft smarta i ändtarmsöppningen

In an earlier publication from our research team, self-reported radiation-induced symptoms were analyzed using modified exploratory factor analysis approach and factor loadings for each symptom, factor-specific factor-loading cutoffs and factor scores were estimated (Steineck et al. https://doi.org/10.1371/journal.pone.0171461)

Appendix S2: Single-item questions on different aspects of self-reported quality of life (QoL) and answer alternatives from the postal questionnaire

English translation	Original Swedish text
**Global quality of life**<br>How has your quality of life been in the last 6 months?<br>1 = No quality of life at all 7 = Best possible quality of life	Hur har Din livskvalitet varit, det senaste halvåret?<br>1 = Ingen livskvalitet alls 7 = Bästa möjliga livskvalitet
**Global physical health**<br>How has your physical health been in the last 6 months?<br>1 = Worst imaginable health 7 = Best imaginable health	Hur har Din kroppsliga hälsa varit det senaste halvåret?<br>1 = Sämsta tänkbara hälsa 7 = Bästa tänkbara hälsa
**Physical strength (condition)**<br>How has your physical strength (condition) been in the last 6 months?<br>1 = No strength 7 = Best imaginable strength	Hur har Din kroppsliga ork (kondition) varit, det senaste halvåret?<br>1 = Ingen ork 7 = Bästa tänkbara ork
**Depressed or feeling sad**<br>Have you felt down or depressed in the last 6 months?<br>1 = Never 7 = All the time	Har Du känt Dig nedstämd eller deprimerad, det senaste halvåret?<br>1 = Aldrig 7 = Hela tiden
**Worry or anxiety**<br>Have you felt worry or anxiety in the last 6 months?<br>1 = Never 7 = All the time	Har Du känt oro eller ångest, det senaste halvåret?<br>1 = Aldrig 7 = Hela tiden
**Psychological wellbeing**<br>How has your mental wellbeing been in the last 6 months?<br>1 = No wellbeing 7 = Best possible wellbeing	Hur har Ditt psykiska välbefinnande varit, det senaste halvåret?<br>1 = Inget välbefinnande 7 = Bästa tänkbara välbefinnande
**Having meaning in life**<br>Has your life felt meaningful in the last 6 months?<br>1 = Never 7 = All the time	Har Ditt liv känts meningsfullt, det senaste halvåret?<br>1 = Aldrig 7 = Hela tiden
**Self-esteem**<br>How has your self-esteem been in the last 6 months?<br>1 = No self-esteem 7 = Best imaginable self-esteem	Hur har Din självkänsla varit, det senaste halvåret?<br>1 = Ingen självkänsla 7 = Bästa tänkbara självkänsla
**Satisfied with sleep**<br>How satisfied have you been with your sleep in the last 6 months?<br>1 = Not at all satisfied 7 = Completely satisfied	Hur nöjd har Du varit med Din situation, det senaste halvåret? Sömn<br>1 = Inte alls nöjd 7 = Helt nöjd
**Satisfied with concentration**<br>How satisfied have you been with your concentration in the last 6 months?<br>1 = Not at all satisfied 7 = Completely satisfied	Hur nöjd har Du varit med Din situation, det senaste halvåret? Koncentration<br>1 = Inte alls nöjd 7 = Helt nöjd
**Satisfied with memory**<br>How satisfied have you been with your memory in the last 6 months?<br>1 = Not at all satisfied 7 = Completely satisfied	Hur nöjd har Du varit med Din situation, det senaste halvåret? Minne<br>1 = Inte alls nöjd 7 = Helt nöjd

AGReMA
A Guideline for Reporting Mediation Analyses

Section/Topic	Item Number	Item Description	Reported on page No
**Title and abstract**			
Title	1	Identify that the study uses mediation analysis	1
Abstract	2	Provide a structured summary of the objectives, methods, results, and conclusions specific to mediation analyses	2–3
**Introduction**			
Background and rationale	3	Describe the study background and theoretical rationale for investigating the mechanisms of interest. Include supporting evidence or theoretical rationale for why the intervention or exposure might have a causal relationship with the proposed mediators. Include supporting evidence or theoretical rationale for why the mediators might have a causal relationship with the outcomes	3–6
Objectives	4	State the objectives of the study specific to the mechanisms of interest. The objectives should specify whether the study aims to test or estimate the mechanistic effects	6
**Methods**			
Study registration	5	If applicable, provide references to any protocols or study registrations specific to the mediation analysis, and highlight any deviations from the planned protocol	NA
Study design and source of data	6	Specify the design of the original study that was used in mediation analyses and where the details can be accessed, supported by a reference. If applicable, describe study design features that are relevant to mediation analyses	7–8
Participants	7	Describe the target population, eligibility criteria specific to mediation analyses, study locations, and study dates (start of participant enrolment and end of follow-up)	7–8
Sample Size	8	State whether a sample size calculation was conducted for mediation analyses. If so, explain how it was calculated	Not conducted
Effects of interest	9	Specify the effects of interest	13
Assumed causal model	10	Include a graphic representation of the assumed causal model including the exposure, mediator, outcome, and possible confounders	12
Causal assumptions	11	Specify assumptions about the causal model	11–12
Measurement	12	Clearly describe the interventions or exposures, mediators, outcomes, confounders, and moderators that were used in the analyses. Specify how and when they were measured, the measurement properties, and whether blinded assessment was used	8–10
Measurement levels	13	If relevant, describe the levels at which the exposure, mediator, and outcome were measured	8–10, 12

(cc) BY-NC-ND

Statistical methods	14	Describe the statistical methods used to estimate the causal relationships of interest. This description should specify analytical strategies used to reduce confounding, model building procedures, justification for the inclusion or exclusion of possible interaction terms, modelling assumptions, and methods used to handle missing data. Provide a reference to the statistical software and package used	10—13
Sensitivity analyses	15	Describe any sensitivity analyses that were used to explore causal or statistical assumptions and the influence of missing data	11, 14
Ethical approval	16	Name the institutional research board or ethics committee that approved the study. Provide a description of participant informed consent or ethics committee waiver of informed consent	8
**Results**			
Participants	17	Describe baseline characteristics of participants included in mediation analyses. Report the total sample size and number of participants lost during follow-up or with missing data	14, Table 1
Outcomes and estimates	18	Report point estimates and uncertainty estimates for the exposure-mediator and mediator-outcome relationships. If inference concerning the causal relationship of interest is considered feasible given the causal assumptions, report the point estimate and uncertainty estimate	15, Table 3
Sensitivity parameters	19	Report the results from any sensitivity analyses used to assess robustness of the causal or statistical assumptions, and the influence of missing data	15, Table S3
**Discussion**			
Limitations	20	Discuss the limitations of the study including potential sources of bias	18—19
Interpretation	21	Interpret the estimated effects considering the study's magnitude and uncertainty, plausibility of the causal assumptions, limitations, generalizability of the findings, and results from relevant studies	16—20
Implications	22	Discuss the implications of the overall results for clinical practice, policy, and science	19—20
**Other information**			
Funding and role of sponsor	23	List all sources of funding or sponsorship for the mediation analysis and the role of the funders/sponsors in the conduct of the study, writing of the manuscript, and decision to submit for publication.	1, 20
Conflicts of interest and financial disclosures	24	State any conflicts of interest and financial disclosures for all authors	1
Data and code	25	Authors are encouraged to provide a statement for sharing data and code for the mediation analysis	2

From: Lee H, Cashin AG, Lamb SE, Hopewell S, Vansteelandt S, VanderWeele TJ, et al. A Guideline for Reporting Mediation Analyses of Randomized Trials and Observational Studies. The AGReMA Statement. JAMA. 2021;326(11):1045–1056. doi:10.1001/jama.2021.14075

AGReMA is designed for articles that report mediation analyses of randomized trials or observational studies.
For more information, visit: agrema-statement.org

(cc) BY-NC-ND

Table S1. Association between suffering from urgency syndrome (in 2006) and being awarded a disability pension (in 2008) in all survivors fulfilling the rigorous inclusion criterion (age <65 years and no earlier disability pension during 2004—2006)

Urgency syndrome	Disability pension	
N=187	Yes (n=7)	No (n=180)
Yes (n=61)	5 (8%)	56 (92%)
No (n=126)	2 (2%)	124 (98%)

Table S2: Adjusted natural direct effect (NDE), indirect effect (NIE), and total effect (TE) of radiation-induced urgency syndrome on disability pension in presence of exposure-mediator interaction (n=187 gynecological cancer survivors). Data on disability pension was obtained from the official register; the table shows the findings of mediation analysis while adjusting for age (in years) , marital status, occupation-based socio-economic group, and number of comorbidities

**Mediator (M)**	**Adjusted relative risk† (95% confidence interval (CI))**			**Proportion mediated‡ (95% CI)**
	**Natural direct effect (NDE)**	**Natural indirect effect(NIE)**	**Total effect (TE)**	
Global quality of life	5.9	1.5	8.6	36%
**Physical aspects**				
Global physical health	5.0	1.5	7.5	39%
Physical strength (condition)	5.0	1.0	5.2	4%
**Psychological aspects**				
Satisfied with sleep	6.1	1.4	8.6	33%
Psychological wellbeing	8.3	1.4	12.0	33%
Satisfied with concentration	11.4	1.6	17.8	38%
Self-esteem	9.4	1.2	11.7	21%
Worry or anxiety	7.9	1.5	11.5	35
Having meaning in life	5.5	1.0	5.6	2%
Satisfied with memory	5.7	1.5	8.7	39%
Depressed or feeling sad	28.2	0.8	22.6	-26%

Estimates of natural direct effect, indirect effect, total effect, and proportion of effect mediated were obtained by using an aspect of self-assessed quality of life as a mediator(M) for the association between urgency syndrome(X) and disability pension(Y) in presence of XM-interaction † Adjusted relative risk with bootstrap bias corrected 95% confidence interval. ‡ Proportion mediated = NDE*(NIE-1)/(NDE*NIE-1), with bootstrap bias corrected 95% confidence interval. §Wald 95% confidence interval. **Bold numbers** indicate a statistically significant effect at 5% level of significance. **NDE** = the contrast between the counterfactual outcome while being exposed and the counterfactual outcome while the same individual was not exposed, mediator assuming value it would have taken while being not exposed. **NIE** = the contrast, having set the *exposure =Yes, exposed* between the counterfactual outcome (mediator assumed whatever value it would have taken at a value of the *exposure =Yes, exposed* and the counterfactual outcome if the mediator assumed whatever value, it would have taken at a reference value of the *exposure =not exposed*).

Table S3: Sensitivity analysis of an unmeasured confounding using mediational E-values

Mediator (M)	Natural direct effect (NDE)†	E-value for NDE	Natural indirect effect(NIE)†	E-value for NIE
**Global quality of life**	1.3 (0.8–2.4)	2.0	**1.6 (1.3–2.0)**	2.5‡
**Physical aspects**				
Global physical health	1.7 (0.8–2.8)	2.8	**1.3 (1.0–1.6)**	2.0
Physical strength (condition)	1.7 (0.9–3.4)	2.8	**1.2 (1.0–1.4)**[§]	1.7
**Psychological aspects**				
Satisfied with sleep	**1.6 (0.8–3.1)**	2.6	**1.3 (1.1–1.7)**	2.0
Psychological wellbeing	**1.7 (1.0–2.5)**	2.9	**1.3 (1.1–1.6)**	2.0
Satisfied with concentration	**1.5 (1.0–2.3)**	2.4	1.2 (1.0–1.4)	1.7
Self-esteem	**1.9 (1.1–5.8)**	3.1	1.2 (1.0–1.3)	1.6
Worry or anxiety	1.6 (0.8–2.7)	2.6	1.1 (0.9–1.4)	1.4
Having meaning in life	1.7 (0.9–3.7)	2.8	1.1 (1.0–1.3)	1.4
Satisfied with memory	**1.9 (1.0–2.8)**	3.2	1.1 (0.8–1.3)	1.3
Depressed or feeling sad	**2.2 (1.3–4.5)**	3.9	1.0 (0.9–1.3)	1.1

†Adjusted relative risk† (95% confidence interval (CI)). E-values for NDE and NIE describes the strength of the confounder–disability pension association and the approximate strength of the confounder–Qol association that, together, would be required to explain away the observed direct and indirect effect, respectively.

‡To completely explain away the ***observed indirect effect*** *(RR=1.6) of global Qol, an unmeasured confounder associated with both global Qol and disability pension with approximate relative risk of 2.5 each, above and beyond the measured confounding, could suffice, but weaker confounding could not. To shift the confidence interval to the null, an unmeasured confounding associated with both global Qol and disability pension with approximate relative risk of 2.5 each, above and beyond the measured confounding, could suffice, but weaker confounding could not.*

Table S4: Adjusted natural direct effect (NDE), indirect effect (NIE), and total effect (TE) of radiation-induced urgency syndrome on disability pension in absence of exposure-mediator interaction (n=247 gynecological cancer survivors). Data on disability pension was obtained from the official register; the table shows the findings of mediation analysis while adjusting for age (in years) , marital status, occupation-based socio-economic group, and number of comorbidities

Mediator (M)	Adjusted relative risk† (95% confidence interval (CI))			Proportion mediated‡ (95% CI)
	Natural direct effect (NDE)	Natural indirect effect(NIE)	Total effect (TE)	
Global quality of life	**1.4 (1.1–2.5)**	**1.3 (1.2–1.6)**	**1.8 (1.1–2.7)**	**54% (36%–177%)**
**Physical aspects**				
Global physical health	**1.6 (1.6–4.3)**	**1.3 (1.1–1.5)**	**2.1 (1.4–3.2)**	**43% (27%–73%)**
Physical strength (condition)	1.6 (0.9–2.2)	**1.2 (1.0–1.3)**	**1.8 (1.0–2.5)**	**29% (14%–89%)**
**Psychological aspects**				
Satisfied with sleep	1.2 (0.8–1.8)	**1.2 (1.1–1.4)**	1.5 (0.9–1.8)	**55% (15%–95%)**§
Satisfied with concentration	**1.6 (1.4–3.6)**	**1.2 (1.1–1.4)**	**2.0 (1.2–2.9)**	**37% (17%–103%)**
Satisfied with memory	**1.9 (1.4–4.3)**	**1.2 (1.1–1.4)**	**2.3 (1.4–3.8)**	**28% (8%–66%)**
Psychological wellbeing	**2.0 (1.6–3.7)**	**1.1 (1.0–1.3)**	**2.2 (1.5–3.5)**	**22% (4%–41%)**
Self-esteem	**1.9 (1.4–3.5)**	**1.1 (1.0–1.3)**	**2.1 (1.4–3.4)**	**21% (2%–48%)**
Worry or anxiety	**1.8 (1.3–3.0)**	**1.1 (1.0–1.3)**	**2.0 (1.3–3.0)**	**21% (5%–56%)**
Having meaning in life	**1.8 (1.2–3.9)**	1.1 (1.0–1.2)	**1.9 (1.2–3.5)**	15% (-1%–55%)
Depressed or feeling sad	**2.4 (2.0–6.4)**	1.0 (0.9–1.2)	**2.5 (1.7–4.3)**	4% (-14%–31%)

Estimates of natural direct effect. indirect effect. total effect. and proportion of effect mediated were obtained by using an aspect of self-assessed quality of life as a mediator(M) for the association between urgency syndrome(X) and disability pension(Y) in absence of exposure-mediator interaction † Adjusted relative risk with bootstrap bias corrected 95% confidence interval. ‡ Proportion mediated = NDE*(NIE-1)/(NDE*NIE-1). with bootstrap bias corrected 95% confidence interval. §Wald 95% confidence interval. **Bold numbers** indicate a statistically significant effect at 5% level of significance. **NDE** = the contrast between the counterfactual outcome while being exposed and the counterfactual outcome while the same individual was not exposed. mediator assuming value it would have taken while being not exposed. **NIE** = the contrast. having set the *exposure =Yes. exposed* between the counterfactual outcome (mediator assumed whatever value it would have taken at a value of the *exposure =Yes. exposed* and the counterfactual outcome if the mediator assumed whatever value. it would have taken at a reference value of the *exposure =not exposed*).
